# Lowering blood pressure by changing lifestyle through a motivational education program: a cluster randomized controlled trial study protocol

**DOI:** 10.1186/s13063-021-05379-2

**Published:** 2021-07-08

**Authors:** Fakir M Amirul Islam, Elisabeth A. Lambert, Sheikh Mohammed Shariful Islam, M. Ariful Islam, Dip Biswas, Rachael McDonald, Ralph Maddison, Bruce Thompson, Gavin W. Lambert

**Affiliations:** 1 School of Health Sciences, Swinburne University of Technology, Hawthorn, VIC 3122 Australia; 2 Organisation for Rural Community Development (ORCD), Dariapur, Narail, Bangladesh; 3 Iverson Health Innovation Research Institute, Swinburne University of Technology, Hawthorn, VIC 3122 Australia; 4 Institute for Physical Activity and Nutrition (IPAN), School of Exercise & Nut. Sci., Deakin University, Burwood, VIC 3125 Australia

## Abstract

**Background:**

High blood pressure is an independent risk factor of cardiovascular disease (CVD) and is a major cause of disability and death. Managing a healthy lifestyle has been shown to reduce blood pressure and improve health outcomes. We aim to investigate the effectiveness of a lifestyle modification intervention program for lowering blood pressure in a rural area of Bangladesh.

**Methods:**

A single-center cluster randomized controlled trial (RCT). The study will be conducted for 6 months, a total of 300 participants of age 30 to 75 years with 150 adults in each of the intervention and the control arms. The intervention arm will involve the delivery of a blended learning education program on lifestyle changes for the management of high blood pressure. The education program comprises evidence-based information with pictures, fact sheets, and published literature about the effects of high blood pressure on CVD development, increased physical activity, and the role of a healthy diet in blood pressure management. The control group involves providing information booklets and general advice at the baseline data collection point. The primary outcome will be the absolute difference in clinic SBP and DBP. Secondary outcomes include the difference in the percentage of people adopting regular exercise habits, cessation of smoking and reducing sodium chloride intake, health literacy of all participants, and the perceived barriers and enablers to adopt behavior changes by collecting qualitative data. Analyses will include analysis of covariance to report the mean difference in blood pressure between the control and the intervention group and the difference in change in blood pressure due to the intervention.

**Discussion:**

The study will assess the effects of physical activity and lifestyle modification in controlling high blood pressure. This study will develop new evidence as to whether a simple lifestyle program implemented in a rural region of a low- and middle-income country will improve blood pressure parameters for people with different chronic diseases by engaging community people.

**Trial registration:**

ClinicalTrials.gov NCT04505150. Registered on 7 August 2020

## Background

High blood pressure is one of the fundamental causes of cardiovascular disease (CVD) and a major cause of disability and death. Raised blood pressure accounts for as many as 10.4 million deaths per year globally [1, 2]. The prevalence of high blood pressure has increased from 87.0 million in 1999–2000 to 108.2 million in 2015–2016 [3]. The estimated number of annual deaths is 1.5 million worldwide, of which 9.4% has been reported to be due to high blood pressure [1]. Modifiable risk factors such as smoking, unhealthy diet, and physical inactivity are shared and established risk factors for CVDs [2, 4]. Different strategies including lifestyle changes such as smoking cessation, diet alteration including a reduction in dietary sodium intake, increased physical activity, and adherence to medication can help in managing high blood pressures and improve health outcomes by decreasing or slowing complications associated with high blood pressure and other CVDs [4–8], but strategies targeting multiple-level or patient-level barriers are effective in controlling high blood pressure [9].

Development and subsequent evaluation of effectiveness for any intervention targeting high blood pressure should include core components comprising baseline participant assessment, educational interventions for participants to acquire adequate knowledge about the etiology and risk factors of hypertension, and modification of lifestyle factors such as participating in regular physical activity, consuming a healthy diet, and smoking cessation [10]. One of the successful models in increasing hypertension awareness, control, and treatment is the Canadian Hypertension Education Program (CHEP), which has been recommended for the development and implementation of healthcare system at the international level [11, 12]. Despite the high prevalence of high blood pressure in low-income and middle-income countries, there are relatively few data on intervention programs from low-income and middle-income countries (LMICs) [13]. Studies also report that a significant proportion of people with high blood pressure are undiagnosed or do not meet targets to control blood pressure both in developed [14] and in LMICs [15, 16]. Among people with known high blood pressure, less than one-third of people can control their high blood pressure with appropriate treatment [13, 17]. Lifestyle modification programs have been shown to be effective in controlling blood pressure in LMICs [18–20].

Bangladesh, a low-income country in South East Asia, is currently confronting an increasing burden of chronic diseases, including high blood pressure [21]. Islam et al. conducted a cross-sectional study among adults age ≥ 30 years in a rural district in Bangladesh that reported the knowledge, attitudes, and practice of diabetes [22] and common eye diseases [23], and found overall knowledge was below average. The study also reported the prevalence and risk factors associated with known and undiagnosed diabetes [24] and self-reported known high blood pressure and newly diagnosed high blood pressure [15]. The study identified that 40% of adults had stage 1 high blood pressure with a cut-off of systolic blood pressure (SBP) ≥130 mmHg or diastolic blood pressure (DBP) ≥80 mmHg or taking antihypertensive [25], of which 82% were previously undiagnosed [15]. Almost 60% of those with high blood pressure also had diabetes [24].

Recently, a community-based cluster randomized controlled trial was conducted in rural communities in Bangladesh, Pakistan, and Sri Lanka. This multicomponent intervention program was conducted for 24 months in 2645 adults with hypertension defined as blood pressure greater than or equal to 140/90 mmHg. The study reported a statistically significant mean reduction of SBP of 5.2 mmHg from the baseline mean blood pressure 146.7 mmHg compared to the usual care in which the reduction was 3.9 mmHg. A similar mean reduction in SBP was observed in a previous cluster randomized clinical trial. Therapeutic lifestyle change intervention plus motivational interviewing intervention for 6 months was associated with SBP reduction of 5.79 mmHg compared to health education intervention only [26]. Relatively small reductions of 2 mmHg in SBP and DBP have been reported to lower the risk of stroke by 14% and 17%, respectively, and the risk of coronary artery disease by 9% and 6%, respectively [27]. The intervention program included home visits by government community health workers for blood pressure monitoring and counseling and the training of physicians [20]. A possible limitation of this intervention program [20] may be that home visits by trained community health workers for blood pressure monitoring and counseling may not be a sustainable and cost-saving approach due to the shortage of qualified workforce and the budget constraints in low-middle-income countries. Application of blended learning education programs to educate patients in managing blood pressure by changing lifestyle and participation in intervention programs as volunteers coordinated by volunteer leaders may be a sustainable approach for lowering blood pressure in the community setting. The goal of this study is to compare a multicomponent intervention program to evaluate its effectiveness for lowering blood pressure among adults with high blood pressure in a rural district in Bangladesh.

Health literacy is a complex and combined perception comprising a range of attributes including available resources of health-related information and an individuals’ intellectual, emotional, social, and personal skills [28]. Health literacy empowers people with skills to improve their health and well-being. Evidence indicates that deficits in health literacy are associated with poorer health outcomes and higher health-related costs at both individual and system levels [29, 30]. Improved health literacy had been reported to be associated with reductions in risk behaviors for chronic disease [31, 32]. However, an extended and often asymptomatic onset and a need for ongoing management, these conditions present people with a sharp and upward learning curve about risks, treatments, and self-care. Self-care, especially in resource-limited settings, an essential dimension of treatment, depends on the ability of systems and providers to teach and patients to learn effective self-management skills [33]. At the individual level, good health literacy is the foundation of successful management and prevention of chronic disease. Health literacy assessment can be used to improve community participation in health, health service planning, public health education, and policy development [34]. In the past decade, much research on the impact of health literacy on health outcomes has been conducted across the globe [32, 35]; however, health literacy tools in rural areas of Bangladesh have not been developed or tested. The European Health Literacy Survey (HLS-Q12) questionnaire has been used to assess health literacy [36]; however, its usefulness to measure health literacy in rural Bangladesh has not been investigated.

### Aim of the study

The aim of this study is to investigate if a health education intervention program lowers high blood pressure.

### Primary objective and hypothesis

#### Objective 1

To assess the effectiveness of a health education program to adopt lifestyle modification in managing systolic and diastolic blood pressure.

#### Hypothesis

A motivational education program to change lifestyle by smoking cessation, taking part in regular physical activities and managing a healthy diet, and receiving a weekly motivational phone call will reduce blood pressure compared to the control group receiving an information booklet and general advice only.

### Secondary objectives related to the intervention

*Objective 1*: To report the difference in the proportion of people adopting regular exercise habits, cessation of smoking, and adopting healthy dietary habits between control and intervention groups

*Objective 2*: To report the general health literacy among the total study participants

*Objective 3*: To report the willingness to receive and pay for a text message system for lifestyle modification

*Objective 4*: To report the perceived barriers and enablers in participating and adopting lifestyle modifications to manage blood pressure

## Methods

Study design: a single-center, cluster randomized control trial (RCT). Control:intervention = 50%:50%

### Duration

Six-month trial

### Participants

The study will be conducted in the Banshgram Union of Narail district, located approximately 200 km southwest of Dhaka, Bangladesh. To understand the location, Bangladesh is a country of over 163 million people divided into 64 districts. Each district is divided into Upazilas (sub-districts), and each Upazila is divided into Unions which consist of 15–20 villages [37]. A cross-sectional study was conducted among 3104 participants of age 30–89 years in 2013 in the Banshgram Union that identified 1242 (40%) participants with hypertension according to WHO guidelines as SBP ≥140 mmHg and/or DBP ≥90 mmHg and/or self-report use of medication for hypertension [15]. Given the intervention of the present study that needs lifestyle modification and more use of a mobile phone, it was decided to limit the age to 75 years as access and use of mobile phones was likely more problematic with older participants [38]. Thus, we chose to limit our upper age to 75 years, leaving 1072 eligible participants for this study. Based on power calculations, we require only 300 participants and expect to recruit the required number of participants without any major difficulty. Inclusion criteria will be based on recent guidelines or definition of hypertension [25]; anyone with SBP ≥130 mmHg and/or DBP ≥80 mmHg and/or self-report use of medication for hypertension will be considered eligible for the current study.

### Sample size

A total of 212 participants (106 per group) will provide 95% power at a significance level of 0.01 to detect a difference of 5 mm/Hg in SBP, assuming a standard deviation of 12 mm/Hg. Assuming loss-to-follow of approximately 40% for various reasons, the required number to recruit is 300 participants (150 per group). This sample size was based on previous research, which showed a decrease in SBP of 5 mm/Hg was associated with a clinically significant reduction in the relative risk of stroke and coronary heart disease events [39].

### Recruitment

Recruitment will be conducted by the investigators of the Organization for Rural Community Development (ORCD) in Bangladesh (www.orcdbd.org). The ORCD is a local non-government organization involved in providing health services to rural people and conducting research. The ORCD investigators, and trained data collectors, will communicate with the potential participants over the telephone or by direct contact. The ORCD participants are not involved in the study design, but they will be involved in the recruitment and conduct of the study. Upon establishment of contact with eligible participants, the baseline data collection will be done through household visits. At household visits, the ORCD investigators and the data collectors will briefly explain the study objectives and their voluntary participation in the project. Interested potential participants will be assessed for eligibility that includes the measurement of blood pressure. Informed written consent will be obtained from all participants. Upon enrolment, a face-to-face interview will be conducted to collect information and record blood pressure from the participants whilst maintaining a social distance. Recruitment will stop once the required number of participants are enrolled. If we are unable to recruit completely from the original cohort, we will expand and recruit participants from outside of this specific cohort but from the exact location. Recruitment from outside of the proposed cross-sectional cohort is not expected to affect the primary objectives. Our source population is over 1000 participants. We do not anticipate any problems in recruiting 300 participants.

### Cluster randomization

Randomization will be performed after the recruitment of all participants and the baseline data collection. The entire 18 villages will be divided into two clusters: one cluster is with nine villages, about 50% of the study location, and the other cluster is with the remaining nine villages. The clusters are the unit of randomization. Each cluster has 150 participants; 10 to 20 participants are from each village based on the village size within a cluster. The intervention and the control group will not be divided into mutually exclusive groups based on risk factors for high blood pressure. This is because (i) this is highly likely that both clusters will have similar risk factors for high blood pressure and (ii) the intervention will be more practical given the nature of the intervention where participants will work as a team formed in different villages.

### Sequence generation

Statistically, a simple random sampling technique (an unbiased coin toss) will be used to select the intervention group. The cluster is the unit of randomness but not the individual participants within clusters. However, within a cluster, we will aim to recruit participants with a male to female ratio equivalent. In that case, the restriction is to recruit one male after recruiting one female participant to make the sex ratio equivalent. Therefore, no random sequence will be generated. However, the complete list of potential percipients with hypertension is available from the previous cross-sectional study [15]. The study location, participants, and their socioeconomic condition can be accessed from the Narail district statistics [40].

### Inclusion criteria

- Participants with clinic blood pressure more than or equal to 130/80 mmHg who are not taking medication
- Participants with controlled blood pressure defined as < 130/80 using antihypertensive medication for a minimum of 6 weeks
- Participants live in Banshgram Union only

### Exclusion criteria

- Aged > 75 years of age
- Pregnant women
- People who have advanced CVDs or have any serious condition that restricts their participation in the study
- Participants will be withdrawn from the study if they are unwilling to continue their participation and withdraw their consent, or any women participants who become pregnant during the study

### Dropout

Participants will be considered as a dropout if they are not willing to continue their participation for any reasons or they withdraw due to health problems or if someone died.

### Implementation of the intervention

#### Intervention

In the intervention arm of 150 participants, the ORCD investigators will form at least ten teams, led by a volunteer leader who will lead the teams and communicate with the project investigator or the doctor if it is necessary. The intervention arm will involve (i) delivering a blended learning education program, including delivering printed materials, and (ii) weekly phone calls to the team leaders. The intervention is an educational program focused on awareness of a healthy lifestyle, including motivation to participate in regular physical activity, community engagement, and keeping frequent contact with health professionals. The chief investigator will keep a record of each team and communicate with the team leaders once every week to remind them to adhere to the intervention program. The weekly monitoring program will continue until the study ends.

The lectures will be delivered to ten teams, comprised of about 15 participants in each team, and will be led by a volunteer team leader using the ORCD’s laptop, overhead projector, and sound system in local clubs, school premises, or participants’ houses. The ORCD investigator, including the physician and an information technology person, will assist with the education programs. Where required, the physician will add interpretations or additional explanations following WHO guidelines and recommendations [41].

#### Control group

The control group will only receive the printed materials at their initial clinic visit.

### Intervention materials (learning materials) for lifestyle modification

The intervention materials will comprise three learning modules: module 1—the effect of high blood pressure on CVDs such as its association with stroke, heart attack and heart failure, kidney diseases, and sexual dysfunction; module 2—managing high blood pressure by managing a healthy weight, taking part in regular exercise, managing stress, consuming a healthy diet including eating of a variety of fruits and vegetables, and avoiding foods high in sodium including raw salt; and module 3—smoking and its associations with cancers and CVDs, and motivation of smoking cessation.

The learning materials will include evidence-based information with pictures, fact sheets, and published literature. The learning materials will be refined following consultation before printing and recording. Ten non-participants, five women and five independent of the study, will be requested to read the learning materials to assess comprehension, wording, and appropriateness. The learning materials will be refined and prepared both in printed version and recorded lectures. The chief investigator will record three modules of about 15 min each, i.e., 45–50 min in total in Bengali language using a video recording software, Camtasia (TechSmith *Camtasia 2019*). Each team leader will be given one Omron blood pressure measuring unit free of cost.

### Interventions that are permitted or prohibited

Participants will receive learning kits and motivational phone calls related to the intervention. The exercise programs will not be supervised but study investigators will facilitate in forming peer groups to take part in various activities as teams. Women will participate as a team of at least five women. The participants will be encouraged not to take part in physical activity alone. In the information sheet provided to the participants, the address of the ORCD and Swinburne’s ethics office will be supplied with information that participants can use to contact if they encounter any serious events. Given the current coronavirus-2019 (COVID-19) situation in the study location (identified as the least affected area in Bangladesh), data collection and intervention delivery will be managed by meeting appropriate social distancing requirements and following government policy using facemask and hand sanitizer to mitigate virus transmission.

### Equipment and blood pressure measurements

Blood pressure will be measured using a calibrated Omron Premium Blood Pressure Monitor Device, Omron HEM-7322 (Australia). Blood pressure will be measured from the right arm with the person sitting upright after 3 min of rest, and a further measurement will be taken following a period of at least 3 min of rest. A third measure will be taken if a major difference is observed between the first two measures and the nearest two measures will be recorded. The two readings will be averaged for SBP and diastolic blood pressure (DBP) separately. Participants will be considered to have high blood pressure if recorded SBP ≥130 mmHg and/or DBP ≥80 mmHg.

### Outcome measures

#### Primary outcome

The primary outcome is the difference in clinical SBP and DBP within and between control and intervention groups. We will report both the absolute difference and the difference in change after follow-up.

#### Secondary outcomes

(i) Psychometric properties of the HLS-EU-Q12 [36] questionnaire which is a 12-item questionnaire and was developed and evaluated in European countries for measuring health literacy. There are no validated tools for measuring health literacy in Bangladesh where HLS-EU-Q12 could potentially be used. However, it is unknown whether all items are appropriate as in Bangladesh. Therefore, assessing the reliability and validity of the HLS-EU-Q12 tool is a secondary outcome. Psychometric properties of the HLS-EU-Q12 [36] will be assessed at baseline only.
(ii) Level of health literacy at baseline. After assessing the psychometric properties, we may come up with all 12 items or remove one or two items. Based on the validated tool, we will estimate the health literacy among the participants with high blood pressure. The outcome measure will be the proportion of people who have different levels of health literacy such as above or below average. The level of health literacy will be estimated at baseline only.
(iii) The proportion of the smoker who smoked at baseline and at 6 months, and the proportion of smokers who intend to quit or reduce smoking within 1 month or 3 months at baseline and achievement after the intervention. Time frame: (1) baseline and (2) immediately after the intervention (6 months).
(iv) The proportion of people who take regular physical exercise and who intend to take part in physical activities. To measure this outcome, the GPAQ [42] tool will be used. The difference in the proportion of participants who take part in regular exercise was measured at baseline and at follow-up. Time frame: (1) baseline and (2) immediately after the intervention (6 months).
(v) The proportion of people who use their own mobile phone, can read SMS, and have the intention to receive and pay for SMS for health information. Time frame: baseline only.
(vi) Perception of and practice in raw salt consumption. The outcome measure is the proportion. Tool: a modified version of Q-MAT [43]. Time frame: (1) baseline and (2) immediately after the intervention (6 months).
(vii) Perceived barriers and enablers in participating and adopting lifestyle modifications to manage blood pressure. This is descriptive information. The software NVivo will be used to investigate if there are any common themes of barriers which could intervene. Time frame: immediately after the intervention (6 months).

### Participant timeline and outcome measures

All participants are recruited from December 2020 to March 2021. Intervention, outcome measures, and timeline are shown in Table 1.

**Table 1:** Time schedule of enrolment, interventions, and outcome measures

	Recruitment and baseline data collection	Intervention and follow-up assessment
Time schedule	December 2020–March 2021	May 2021–November 2021
Parameter assessments		
(i) Blood pressure	√	√
(ii) Participant information including age, gender, level of education, occupation, and self-reported medical history	√	
(iii) Health literacy data using the shorter version of the European Health Literacy Survey Questionnaire HLS-EU-Q12 [36]	√	
(iv) Smoking cessation motivation questionnaire (Q-MAT) [43]	√	√
(v) Global Physical Activity Questionnaire (GPAQ) [44, 45]	√	√
(vi) Dietary motivation questionnaire, which is a modified version of the smoking cessation motivation questionnaire (Q-MAT)	√	√
(vii) Information related to the use of a mobile phone in managing blood pressure	√	
(viii) Qualitative data on perceived barriers and enablers in changing lifestyle in managing blood pressure	√	√
(ix) Information on willingness to adopt the lifestyle change activities		√
(x) Information on willingness to continue the activities after the intervention		√
(x) Information on the effectiveness of the intervention components		√

Data analysis and manuscript writing from the baseline data from March 2021 to October 2021 (until follow-up assessments)

### Participants and collection of qualitative information

A nested qualitative study will be conducted within the trial design. Semi-structured in-depth interviews will be used to capture the perspectives and experiences of the project and perceived barriers and enablers faced to change lifestyle activities in managing blood pressure. Data will be collected at the beginning of the study and at the end of the program.

#### Participants

We will select 25 participants: 20 from the intervention arm and five people involved in the research or are aware of local health systems and aware of barriers and opportunities to manage health and well-being in the community. Firstly, we will randomly select four villages from the nine villages of the intervention group. Secondly, we will select five participants: at least two women, one farmer, one employee, and one business person. The proposed choice will represent gender and occupation. This is expected that the proposed selection will also represent the education level. Although the sample is not entirely random, overall, it will represent the intervention group. Other than the study participants, the five people will include one physician who will help deliver blended lectures, the study coordinator, one retired bank officer, the principal of the ORCD college, and a community leader. Previous studies have demonstrated that in this setting 25 participants would be acceptable [46, 47].

#### Qualitative data collection

Two investigators will conduct semi-structured interviews—the chief investigator from Australia through Zoom and another investigator from the ORCD at the study location. A set of open-ended questions will guide the interviews. Through the interview, topics will be covered on the current barriers of the intervention program. The format will allow for additional questions and issues to be covered as they arise throughout the interview. The responses by the participants will be recorded, but their identification will not be disclosed. The questions will include:

Q1: Could you please tell me how easy or difficult it is to access health services to manage chronic diseases, including hypertension?

Q2: What do you recommend for overcoming the problems of access to appropriate health services in your locality?

Q3: To attend regular exercise programs, what are the barriers to participation?

Q4: What would you recommend for overcoming the barriers to lead a healthy life?

Q5: Currently, more than 40% of adults smoke. What do you consider the barriers to quitting smoking?

Q6. What would you recommend for quitting smoking?

### Statistical analyses

Although the intervention will be conducted as clusters, data will be collected as individuals within clusters and the effect of the intervention will be measured based on individual’s data. Since the randomization is based on clusters, there might occur differences in baseline characteristics of participants. The baseline characteristics for the intervention and the control group will be examined using the chi-square test or Fisher’s exact test for categorical variables and the two-sample *t-*test or Mann-Whitney U test for continuous variables depending on the distributions of the data.

The effect size will be the mean difference in the primary outcome. The comparison will be made between differences for control and intervention groups after the follow-up. More specifically, it could be shown as the difference between DifCon and DifInt. DifCon equals the difference between baseline and follow-up for the control group, and DifInt equals the difference between baseline and follow-up for the intervention group. Such comparison will give the actual effect size due to the intervention. Generalized linear model (GLM) will be used for analytical purpose, and the models will be adjusted for factors which are different between control and intervention groups at baseline. For the secondary outcomes (motivation to smoking cessation, change in dietary patterns, and physical activity), chi-square tests will be used to detect any associations in the outcome variables across gender, age categories, level of education, and socioeconomic status. The difference in proportion with 95% confidence interval between control and intervention groups will be computed using the test of proportion, and odds ratios (OR) with 95% confidence interval will be computed using logistic regression techniques. The models will be adjusted for covariates as appropriate.

Rasch analysis using RUMM30 software will be used to investigate the psychometric properties of the Health Literacy tool and to compute the overall score from the 12-item HLS-Q12 tool. Individual item, as well as the overall score from all items combined, will be compared for gender, age group, level of education, occupation, and SES using chi-square, *t*-tests, or ANOVA as it is appropriate.

### Data management, monitoring, and storage

The chief investigator will communicate with the ORCD investigators at least 3 to 4 times per week to monitor the study to verify the validity, precision, and completeness of data and that the safety and rights of participants are protected. In addition, the CI will respond to any urgent issues as soon as possible when contacted. Data will be collected initially on paper and then will be entered into MS Excel using the ORCD laptop computer. Five percent of the entire data will be entered twice by independent data entry operators to assure accuracy. The CI will randomly check 10% of all participants entered to check the quality of data entry. Frequency and range will be examined to identify outliers, and those will be re-checked at the field level if needed.

After data collection, the questionnaires will be stored at the ORCD’s locked cabinet after data entry. For data transfer, data will be stored in Australia’s Academic and Research Network (*AARNet*) CloudStor for access by the chief investigators. Identification of the participants will be coded, and their records will be available only to the investigators.

The investigators will meet once in 3 months to evaluate the intervention.

### Ancillary and post-trial care

This intervention is aimed to improve lifestyle behaviors and reduce high blood pressure, offering benefit to participants. One Omron blood pressure measuring unit will be given to each group of 15 participants to aid them in monitoring blood pressure. The machines will be supplied to 20 team leaders who will volunteer to use the apparatus by the participants and other community members. After the intervention, the blood pressure devices will not be taken back, which may assist individuals to monitor blood pressure in the future. The participants will have contact with the ORCD. As they will provide care during the intervention, they will be suggested to keep the contact information of the ORCD physician to consult any health issues after the trial is completed. In the consent information, participants will be informed that their participation/non-participation will not give them an advantage or disadvantage in relationship to ORCD or local health authorities.

### Auditing

The study has received ethical clearance from the Swinburne University Human Research Ethics Committee. The ethics office has the option to audit any studies, including the current study. The study is also expected to be audited by the Civil Surgeon of the Narail district.

### Access and data sharing

Data will be kept on a secured One Drive for an indefinite period. De-identified data may be used for teaching purposes as outlined in the Participant Information and Consent form. Data will be analyzed as summary statistics or pooled group data without disclosing the identification of the participants. De-identified data will be posted on an appropriate open access repository if it is required by any open access journal. The participants will be informed about the privacy of data transfer.

Data will be shared upon request and will be made available after 2 years of the study.

### Confidentiality

The research team will know the participants’ names. However, data will be used using de-identified serial number. This is an intervention program where the researchers will need to keep in contact with the participants using their names and mobile numbers. This will be made clear with the participants at the time of consent collections that we will keep their contact, but it will not appear in any analyses or publications. Their privacy will be maintained by not disclosing their identity to any third persons.

## Discussion

Lifestyle and behavioral changes have been shown to be effective to lower blood pressure and decrease or slow down the development of hypertension-related CVDs [4–7]. Due to the increasing prevalence of hypertension and inadequate health facilities, especially in low- and middle-income countries [3], managing blood pressure by changing lifestyle could be the most sustainable option. An intervention study using automated self-management calls and home blood pressure monitoring conducted in Honduras and Mexico [48] reported a significant decrease in blood pressure. Many previous studies to manage blood pressure by lifestyle changes have been conducted by health professionals, nurses, or health technicians [18, 20]. Our intervention program consists of delivering a blended learning education and training program, which is expected to be a more sustainable approach due to the limited resources in LMICs. Another component of our intervention is the engagement of community volunteers. The study coordinator will communicate with the team leaders who will lead various lifestyle modification programs within and across the teams. The current intervention is expected to be more sustainable compared to other studies in which health services have been provided by nurses and other health professionals at individual levels [48, 49], which are anticipated to be more resource-involved intervention programs.

This study will develop new evidence as to whether a simple lifestyle program implemented in a rural region of a low- and middle-income country will improve blood pressure parameters for people with different chronic diseases, namely CVD and diabetes, by engaging community people. The study will also report the proportion of participants who will change their smoking status, will take part in physical activities, and modify dietary patterns in managing blood pressure. Intervention through motivational counseling has reported a significant increase in quitting smoking [50], reduction in blood pressure, active participation in physical activities, and changing dietary patterns [51, 52]. Therefore, our results may inform future studies in similar settings to test whether this program can be successfully implemented into a wider non-communicable disease prevention and management program. The study will also report the status of health literacy in rural people in Bangladesh. The findings will inform the future design and conducting of appropriate interventions that could be used to deliver long-term programs. Previous studies, for instance, have demonstrated some benefit in the use of mobile phone interventions for diabetes management [53, 54], hypertension [55], and other healthcare services [56]. However, most of these programs have been small-scale pilot projects and not transformed into a community program. Although this is a cluster RCT design to lower blood pressure by modifying lifestyle, we have incorporated questions to collect data on mobile use among participants and their willingness to receive and pay for receiving a text message system for receiving health information.

## Abbreviations

ORCD: Organization for Rural Community Development
CVD: Cardiovascular disease
LMICs: Low- and middle-income countries
HLS: Health Literacy Survey
Q-MAT: Smoking cessation motivation questionnaire
GPAQ: Global Physical Activity Questionnaire
RCT: Randomized controlled trial
ANCOVA: Analysis for covariance
AARNet: Australia’s Academic and Research Network
